# BCG strain S4-Jena: An early BCG strain is capable to reduce the proliferation of bladder cancer cells by induction of apoptosis

**DOI:** 10.1186/1475-2867-10-21

**Published:** 2010-06-29

**Authors:** Katja Schwarzer, Martin Foerster, Thomas Steiner, Inge-Marie Hermann, Eberhard Straube

**Affiliations:** 1 Institute of Medical Microbiology, Friedrich-Schiller-University, Jena, Germany; 2 Clinic for Internal Medicine I, Pneumology, Friedrich-Schiller-University, Jena, Germany; 3 Department of Urology, Friedrich-Schiller-University, Jena, Germany; 4 Electron Microscopy Centre, Friedrich-Schiller-University, Jena, Germany

## Abstract

**Background:**

Intravesical immunotherapy with *Mycobacterium bovis *bacillus Calmette-Guérin has been established as the most effective adjuvant treatment for high risk non-muscle-invasive bladder cancer (NMIBC). We investigated the differences between the S4-Jena BCG strain and commercially available BCG strains. We tested the genotypic varieties between S4-Jena and other BCG strains and analysed the effect of the BCG strains TICE and S4-Jena on two bladder cancer cell lines.

**Results:**

In contrast to commercially available BCG strains the S4-Jena strain shows genotypic differences. Spoligotyping verifies the S4-Jena strain as a BCG strain. Infection with viable S4-Jena or TICE decreased proliferation in the T24 cell line. Additionally, hallmarks of apoptosis were detectable. In contrast, Cal29 cells showed only a slightly decreased proliferation with TICE. Cal29 cells infected with S4-Jena, though, showed a significantly decreased proliferation in contrast to TICE. Concordantly with these results, infection with TICE had no effect on the morphology and hallmarks of apoptosis of Cal29 cells. However, S4-Jena strain led to clearly visible morphological changes and caspases 3/7 activation and PS flip.

**Conclusions:**

S4-Jena strain has a direct influence on bladder cancer cell lines as shown by inhibition of cell proliferation and induction of apoptosis. The data implicate that the T24 cells are responder for S4-Jena and TICE BCG. However, the Cal29 cells are only responder for S4-Jena and they are non-responder for TICE BCG. S4-Jena strain may represent an effective therapeutic agent for NMIBC.

## 1. Background

In non-muscle-invasive bladder cancer (NMIBC) patients with a high-risk for recurrence an immediate chemotherapy should be followed by adjuvant therapy with *Mycobacterium bovis *bacillus Calmette-Guerin (BCG) [1]. The mechanism by which BCG is effective in the treatment of bladder cancer remains still controversially discussed, yet. BCG therapy results finally in a local immune response characterised by cytokine expression of bladder cells [2]. In addition, influx of granulocytes and mononuclear cells into bladder wall has been observed [3]. Several studies indicate a high survival rate and persistence of BCG in the bladder wall after intravesical BCG-treatment [4,5]. Several in vitro studies demonstrated a direct effect of BCG on bladder cancer cell lines in the absence of immune mechanisms [6]. These effects included cell cycle arrest [7], inhibition of cell proliferation [8-10] as well as apoptosis [11]. The BCG strain applied for NMBIC therapy showed different effects on bladder cancer cell lines.

### History of S4-Jena

The BCG strain S4-Jena applied in this study was brought from Gothenburg to Jena in 1950. In contrast to vaccines prepared from other BCG strains, S4-Jena showed no significant side effects such as systemic infections in neonates [12]. The S4-Jena strain was introduced into a multicentre clinical trial for intravesical adjuvant therapy of bladder cancer from 1988-1991 in 7 hospitals. 217 patients with a tumour stage TaG1-2/T1G1-3 were included. Recurrence-free survival was defined as the time from initial transurethral resection to either recurrence of the tumour. It was shown that 80.2% of the patients had no recurrence within the follow-up period (16.4 months). Side effects like dysuria (41.9%), a conspicuous pathological urinary-status (31.8%) and fever (16.1%) were reported. Based on this study the S4-Jena strain was licensed for bladder cancer therapy in east Europe in 1990.

Regarding the results of this study we focussed in this work on the difference between the S4-Jena BCG strain and commercially available BCG strains. Additionally, we investigated the effects of S4-Jena strain on bladder cancer cell lines in comparison to TICE strain.

## 2. Methods

### 2.1 Propagation of strain

The S4-Jena strain was propagated and harvested by the original method published by Berger in 1953 [13]. The BCG strain S4-Jena was typed via spoligotyping [14] as a *Mycobacterium bovis *ssp. Bacilli Calmette Guerin by the National Reference Centre (NRC) for *Mycobacteria *in Borstel, Germany. The commercially produced BCG strains TICE (Apogepha, Germany), RIVM (Medac, Germany) and Connaught (Cytochemia, Germany) were available as lyophilisates. All BCG strains were used in a concentration of 2 × 10^8^bacteria/ml.

### 2.2 Cell lines

Two human bladder cancer cell lines T24 (ACC376) and Cal29 (ACC515), obtained from German Collection of Microorganisms and Cell Cultures (DSMZ, Germany) were used in this study. Cell lines were cultured as monolayer in D-MEM medium (GIBCO, Germany) supplemented with 10% foetal bovine serum (GIBCO, Germany) at 37°C in an atmosphere with 5%CO_2_.

### 2.3 Pulsed-field gel electrophoresis (PFGE)

DNA preparation was performed as described previously by Slutsky et al, we used VspI as restriction enzyme (HYBAID, Germany) [15]. In short, PFGE (CHEF-DR^®^II, BIO-RAD Lab., USA) was performed in 1% agarose for 22 h at 6 V/cm, pulse times 1-40 s, temperature 15°C. The PFGE pattern was stained with 0.5 μg/ml ethidium bromide (Sigma-Aldrich, USA).

### 2.4 Cell proliferation assay

Cells (1×10^6^/100 μl) were incubated at 37°C with 5% CO_2 _for 24 h. Then they were infected with 10 μl of a 1 × 10^8^/ml CFU BCG S4-Jena or TICE strain. Proliferation was tested by a micro plate-based assay (WST-1, Roche, Germany) that measures metabolic activity using tetrazolium salts [16].

### 2.5 Scanning electron microscopy

Cells were seeded out on sterilized coverslips in 24-well plates (1 × 10^6^/ml), incubated at 37°C and 5%CO_2 _for 24 h and infected with 100 μl of a 1 × 10^8^/ml CFU S4-Jena and TICE strains. After different incubation times (12 h and 24 h) the cells were fixed with 2.5% glutaraldehyde in 0.1 M sodium cacodylate buffer (pH 7.4) at room temperature (RT) for 30 min. Cells were dehydrated in rising ethanol concentrations followed by critical point drying and gold sputter coating in a BAL-TEC-SCD-005 Sputter Coater (BAL-TEC, Germany). The cells were examined using a scanning electron microscope LEO-1450VP (ZEISS, Germany) at 15 kV.

### 2.6 Apoptosis detection by laser scanning microscopy

Cells were seeded out in eight-well Lab-TekTM chamber (1 × 10^4^/ml), incubated at 37°C and 5% CO_2 _for 24 h and infected with 10 μl of 1 × 10^8^/ml CFU S4-Jena and TICE strains. After 24 h infection time, cells were assayed for phosphatidylserine (PS) using the Annexin V FITC-Kit (Invitrogen, Germany). Afterwards, activated caspases-3/7 was visualised using MagicRed™ (Immunochemistry, USA). We used both tests according to manufacturer's directions. The samples were observed under a fluorescence microscope LSM5 EXCITER (ZEISS, Germany).

### 2.7 Statistical analysis

Statistical analysis was performed using parametric Mann-Whitney U-tests for data with a normal distribution. A value of p < 0.05 was considered as significant (*), a value of p < 0.01 was considered as highly significant (**). Data obtained are expressed as mean ± standard deviation (SD).

## 3. Results

### 3.1 Genotypic characterisation of the BCG S4-Jena strain

**Figure 1:**
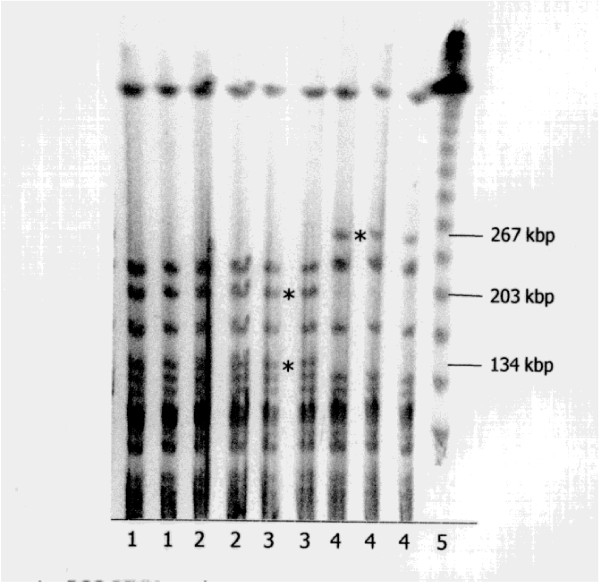
**Genotypic characterization of different BCG strains as analyzed by PFGE**. Lane 1-2: RIVM strain, Lane 3-4: TICE strain, lane 5-6: Connaught strain, 7-9: S4-Jena strain. Lane 10: λ ladder PFG Marker (BioLabs, Frankfurt, Germany).

Using pulsed-field gel electrophoresis we found genotypic varieties between the different BCG strains tested. Genomic DNA was isolated from BCG strains RIVM, TICE, Connaught Canada and S4-Jena. The results in Fig.1 show the different DNA polymorphisms among the kinships. The analysis of the S4-Jena strain showed a fragment with a size of 267 kbp. In contrast, the TICE, RIVM and Connaught strains did not show this fragment, but two smaller ones of 203 kbp und 134 kbp size. However, these both fragments were not detectable in the S4-Jena strain.

### 3.2 Effect of BCG on the proliferation rate of bladder cancer cell lines

**Figure 2:**
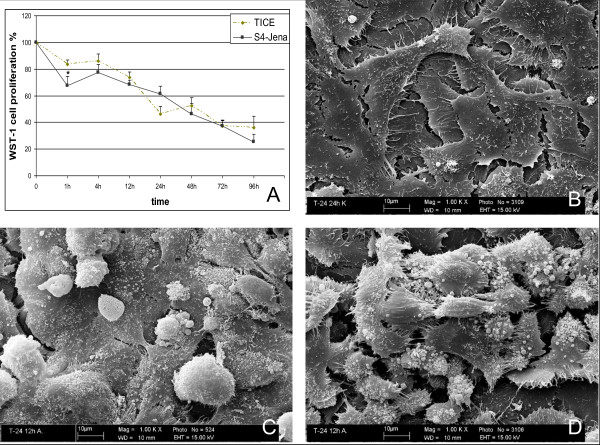
**A: Cell proliferation of T24 cells after infection with BCG S4-Jena and TICE**. Proliferation was measured by WST-1. Results are reported as percentage inhibition of cell proliferation, where the optical density value from untreated cells was set as 100% of proliferation. Test was performed eight times by duplicate measurements. B-D: Effects of BCG strain S4-Jena and TICE on the cell membrane in T24 cells using scanning electron microscopy. Untreated T24 cells are showing a normal surface (B). Infection with TICE (C) and S4-Jena (D) strain led to morphological changes in the cells after 12 h.

T24 and Cal29 cell lines showed different patterns of proliferation after infection with viable BCG. Proliferation of T24 cells decreased during the total incubation period with S4-Jena or TICE (Fig.2A). Only 36% (TICE) and 29% (S4-Jena) of the cells infected with BCG proliferated after 96 h compared to the uninfected control.

**Figure 3:**
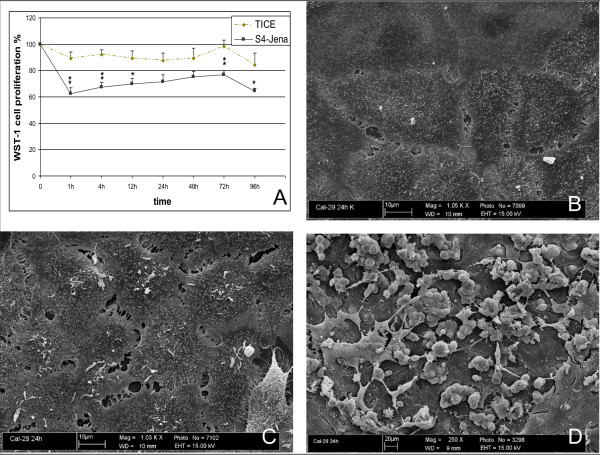
**Cell proliferation of Cal29 cells after infection with BCG S4-Jena and TICE**. Proliferation was measured by WST-1. Results are reported as percentage inhibition of cell proliferation, where the optical density value from untreated cells was set as 100% of proliferation. Test was performed eight times by duplicate measurements. B-D: Effects of BCG strain S4-Jena and TICE on the cell membrane in Cal29 cells using scanning electron microscopy. Untreated Cal29 cells are showing a normal surface (B). Cal29 cells treated with TICE strain for 12 h (C) show a similar appearance as untreated cells. Infection with S4-Jena strain led to morphological changes in the cells after 12 h (D).

In contrast to T24, the Cal29 cells showed only a slightly decreased proliferation of 84-98% compared to the control between 1 h and 96 h after infection with TICE strain (Fig.3A). Cells infected with S4-Jena, however, showed a significantly decreased proliferation (62-77%) in contrast to TICE.

### 3.3 Effect of different BCG-strains on morphology of bladder cancer cell lines

As visualised by electron microscopy both the TICE strain and the S4-Jena strain showed similar effects on the T24 cells (Fig.2B-D). After 12 h the majority of cells exhibited a rounded morphology and detached from the base layer after infection with the strains compared to untreated cells. Additionally, membrane blebbing appeared on the surface of the cells. These effects were more pronounced after infection with S4-Jena than with TICE BCG.

In contrast, the TICE infection had no effect on the morphology of the Cal29 cells even after 24 h of culture. Differing from TICE, the S4-Jena strain led to clearly visible morphological changes also in Cal29 cells even after only 4 h (Fig.3B-D). Again, the infected cells adopted a spherical shape, separated from the united cell structure, and completely lost their adherence. Some cells formed apoptotic membrane blebs. In some areas only cell fragments were visible.

### 3.4 Apoptosis induction by BCG infection

**Figure 4:**
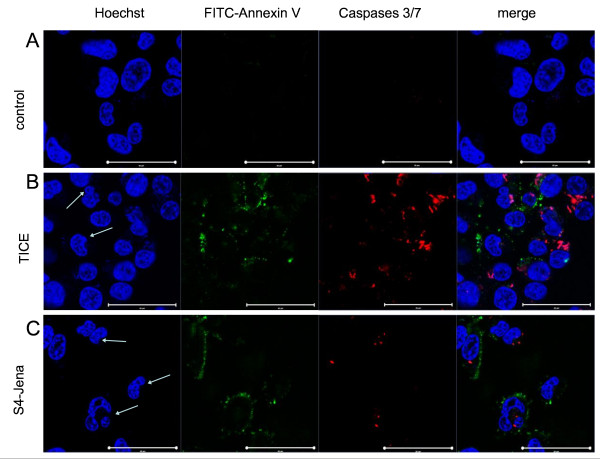
**Apoptotic cell death in T24 cells infected with BCG**. T24 cells were infected with BCG TICE (B) or S4-Jena (C) strain compared to uninfected control cells (A) for 24 h. Cells were stained with Annexin V (green), Caspases 3/7 (red) and Hoechst (blue). Arrowheads show nucleus fragmentation. Scale bars: 50 μm.

**Figure 5:**
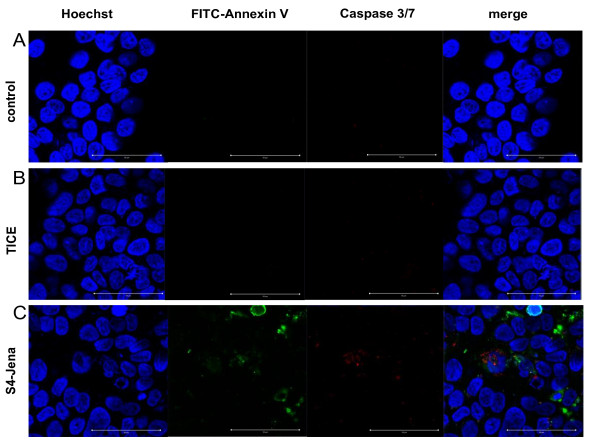
**Apoptotic cell death in Cal29 cells infected with BCG**. Cal29 cells were infected with BCG TICE (B) or S4-Jena (C) strain compared to uninfected control cells (A) for 24 h. Cells were stained with Annexin V (green), Caspases 3/7 (red) and Hoechst (blue). Scale bars: 50 μm.

We used different immunohistochemical tests to detect apoptosis in bladder cancer cell lines after infection with BCG. Apoptosis is a physiological mode of cell death defined by both morphological (e.g. nucleus fragmentation, apoptotic bodies) and biochemical criteria (e.g. activation of caspases). As shown in fig.4, fragmentation of the nucleus (arrowhead) can be detected by staining with Hoechst (blue) in T24 cell after infection with TICE (B) as well as with BCG S4-Jena (C) after 24 h. Activated caspases 3/7 could be shown after infection of T24 cells with both strains. The exposure of PS on the plasma membranes of T24 cells was induced after infection with both strains. Furthermore, infection with S4-Jena led to a lower number of adherent T24 cells, implicating that the majority of cells had detached. In contrast, the Cal29 cells showed only after infection with S4-Jena marginal caspases 3/7 activation and exposure of PS on the plasma membrane (fig.5 C). The infection of Cal29 cells with TICE strain had no influence on caspases 3/7 or PS exposure (fig.5 B).

## 4. Discussion

Intravesical installation of BCG as adjuvant treatment is well established and has been part of routine treatment for patients with superficial bladder cancer for more than 30 years [1,17,18]. The mechanisms by which BCG affects the growth of superficial transitional bladder cancer are still unclear. 30% of bladder cancers are BCG resistant and the long-term durability of the response to BCG is still limited [19]. For the present study we reactivated the S4-Jena strain. We compared S4-Jena with BCG strains which are commercially available for bladder cancer therapy. Additionally, we analysed the effects of S4-Jena and Tice on two different bladder tumour cell lines.

The genotypic varieties between different BCG strains were tested. In this study we compared the commercially available BCG strains RIVM, TICE, Connaught Canada with the S4-Jena strain. Regarding the DNA polymorphisms a clear difference between S4-Jena and the other strains was detectable. The restriction fragment pattern differed in three bands. The S4-Jena strain showed a loss of two bands (203 kbp und 134 kbp) in comparison to the other strains due to the loss of a restriction site. This is confirmed by the occurrence of a 267 kbp band that is not present in the commercial strains. This is an evidence that the S4-Jena strain belongs to the family of BCG strains. Variations of two to three bands have been observed in strains of some species when they are cultured repeatedly over time [20]. These results agree with the conclusion of Brosch et al, who classify the RIVM, TICE and Connaught in a later BCG daughter strain group (DU2 groupIV, Δint) characterized by a large number of deletions [21]. S4-Jena can be allocated in the early DU2 group II, Δint, because it is derived from the Sweden strain. This group shows a low number of deletions.

The second major aspect of this study was a functional characterisation of S4-Jena as a potential agent for adjuvant therapy. As the three commercially available BCG strains are genetically closely related, only one strain was used for further comparative experiments with the S4-Jena strain. The TICE strain was chosen because it belongs to the most comprehensively researched strains. Many studies [3,22,23] assume that BCG infection causes a systemic immunological reaction. In the present study we demonstrated that BCG bacteria even have various direct effects on two different bladder cancer cell lines regarding proliferation and morphology.

A major difference between the two BCG strains was seen in the reduction of cell proliferation. While TICE only reduced proliferation in T24 cells, the S4-Jena strain led to reduced proliferation in both cell-lines tested. Sasaki et al showed a significant suppression of proliferation in T24 cells after infection with the Tokyo-172 strain (DU2 groupI [21]) for 96 h and 120 h [9]. In comparison to these data, in the present study inhibition of proliferation by 40-50% was detectable already after 24 h in T24 cell. Zhang et al described that the Connaught strain (DU2 groupIV, Δint [21]) inhibits dose-dependently the proliferation of bladder cancer cells of various grades [8]. Interestingly, the Connaught strain could also enhance growth in the SD and RT4 cell lines. Rajala et al analysed five BCG strains (Pasteur, Connaught, RIVM, TICE, Evans) for cytostatic activity on bladder cancer cells [10]. They showed that BCG inhibited cell growth and this effect was concentration-dependent in various strains including TICE. In 120 h cultures the BCG strain TICE has a slightly weaker cytostatic effect than the other four strains. Pryor et al have also found that the Pasteur strain exerts a direct, antiproliferative effect on bladder tumour cells in the absence of immune mechanisms. The differences between the various studies may be due to the use of different doses and strains of BCG and different cell lines [6].

Our study clearly shows that the predominant effect of BCG S4-Jena on the bladder cancer cell lines is a reduction of proliferation. In a similar pattern to the effect on the proliferation TICE infection showed no morphological effects on Cal29 cells, but on T24 cells. In contrast, S4-Jena induced morphological changes in both cell lines. The affected cells adopted distinct morphological changes like spherical shape and cell membrane blebbing. They separated from the united cell structure and completely lost their adherence. These are typical hallmarks of apoptosis [24]. Chen et al demonstrated that the TICE strain decreased the viability of bladder cancer cell lines, but did not induce apoptosis via DNA-laddering and caspase-3 activation [7]. Sasaki et al indeed showed an inhibition of proliferation in T24 cells by Tokyo-172 strain, but also concluded that it was not mediated by apoptosis, again employing DNA-laddering after 120 h of incubation [9]. Saitoh et al showed that Tokyo-172 significantly decreased cell viability and induced apoptosis after 120 h in both cell lines [11]. We expanded the methods for apoptosis detection in T24 and Cal29 cells after infection with TICE and S4-Jena strains by morphological features like membrane blebbing and retraction from neighbouring cells. In contrast to Chen et al we could detect caspases 3/7 activation, fragmentation of nucleus and additionally phosphatidylserin-flip after 24 h of BCG infection in T24 cells [7]. The appearance of phosphatidylserine (PS) on the outer leaflet of the cell membrane is one of the earliest indications of apoptosis [24]. In contrast, only S4-Jena but not TICE showed caspases 3/7 activation and PS flip in Cal29 cells supporting our proliferation data. Thus we conclude that the inhibition of proliferation is mediated by an induction of apoptosis. Finally, the data implicate that the T24 cells are responder for S4-Jena and TICE BCG. However, the Cal29 cells are only responder for S4-Jena and they are non-responder for TICE BCG.

## 5. Conclusion

Both tested strains act directly on tumour cells inhibiting the proliferation in T24 cells. Since Cal29 cells were affected by the S4-Jena strain but not by TICE, it can be assumed that not all bladder tumours are susceptible to every BCG strain. This indicates the need for evaluation of susceptibility of bladder tumours to different BCG strains before the adjuvant BCG therapy is initiated. The S4-Jena strain is supposed to be an effective therapeutic agent.

Our study shows clearly that the predominant effect of BCG S4-Jena on the bladder cancer cell lines is a reduction of proliferation that is most likely mediated by apoptosis.

Additionally, our data support the hypothesis of Brosch et al that earlier BCG strains e.g S4-Jena might be superior therapeutic agent than the now common commercially available strains [21].

## Abbreviations

BCG: *Mycobacterium bovis *bacillus Calmette-Guérin; LSM: laser scanning microscopy; NMIBC: non-muscle-invasive bladder cancer; PFGE: pulsed field gel electrophoresis; PS: phosphatidylserine

## Competing interests

The authors declare that they have no competing interests.

## Authors' contributions

KS was responsible for conception and design, acquisition, analysis and interpretation of data, drafting of the manuscript, critical revision of the manuscript for important intellectual content, and statistical analysis. MF was responsible for drafting of the manuscript and critical revision of the manuscript for important intellectual content. IMH was responsible for acquisition of data. TS was responsible for analysis and interpretation of data and critical revision of the manuscript for important intellectual content. ES was responsible for drafting of the manuscript, obtaining funding, administrative, technical, or material support and supervision.

All authors read and approved the final manuscript.
